# Isolation and identification of endophytic actinobacteria from *Citrullus colocynthis* (L.) Schrad and their antibacterial properties

**DOI:** 10.1186/s12934-022-01936-9

**Published:** 2022-10-10

**Authors:** Aram R. Ali, Yadollah Bahrami, Elham Kakaei, Sara Mohammadzadeh, Sasan Bouk, Nastaran Jalilian

**Affiliations:** 1 Department of Medical Biotechnology, School of Medicine, Kermanshah University of Medical Sciences, Kermanshah, Iran; 2 Medical Biology Research Center, Kermanshah University of Medical Sciences, Kermanshah, Iran; 3 Department of Medical Biotechnology, School of Medicine, College of Medicine and Public Health, Flinders University, Adelaide, SA 5042 Australia; 4 Forests and Rangelands Research Department, Kermanshah Agricultural and Natural Resources Research and Education Center, (AREEO), Kermanshah, Iran

**Keywords:** Endophytic actinobacteria, *Citrullus colocynthis*, *Streptomyces*, *Nocardiopsis*, Antibacterial resistance, Antibacterial activity, Natural products, Bioactive compounds, Antibiotic resistance, 16S rRNA gene, Secondary metabolite

## Abstract

**Background:**

Antibiotic resistance poses a major threat to human health globally. Consequently, new antibiotics are desperately required to discover and develop from unexplored habitats to treat life-threatening infections. Microbial natural products (NP) are still remained as primary sources for the discovery of new antibiotics. Endophytic actinobacteria (EA) which are well-known producers of bioactive compounds could provide novel antibiotic against pathogenic bacteria. This research aimed to isolate EA from the *Citrullus colocynthis* plant and explore the antibacterial properties of their metabolites against pathogenic bacteria.

**Results:**

The healthy samples were collected, dissected and surface-sterilized before cultured on four different selection media at 28 °C. Six endophytic actinobacteria were isolated from *Citrullus colocynthis* plant. They were taxonomically classified into two family namely *Streptomycetaceae* and *Nocardiopsaceae,* based on colony morphological features, scanning electron microscope analysis and molecular identification of isolates. This is the first report on the identification of EA form *Citrullus colocynthis* and their antibacterial activity. The strains generated a chain of vibrio-comma, cubed or cylindrical shaped spores with indenting or smooth surfaces. Three of those were reported as endophytes for the first time. The strain KUMS-C1 showed 98.55% sequence similarity to its closely related strains which constitutes as a novel species/ strain for which the name *Nocardiopsis colocynthis* sp. was proposed for the isolated strain. Five isolated strains had antagonist activity against *S. aureus*, *P. aeruginosa*, and *E. coli*. Among those, stain KUMS-C6 showed the broadest spectrum of antibacterial activity against all test bacteria, whereas the strain KUMS-C4 had no antibacterial activity.

**Conclusions:**

NPs have a long history of safe and efficient use for development of pharmaceutical products. Our study highlights that *Citrullus colocynthis* is an untapped source for the isolation of EA, generating novel and bioactive metabolites by which might lead to discovery of new antibiotic(s). This study reveals the future of new antibiotic developments looks bright against multi-drug resistance diseases by mining under- or unexplored habitats.

## Introduction

Antibiotics have given protection against life-threatening bacterial infections, and increased life expectancy for almost a century. However, overuse/misuse and indiscriminate use of antibiotics in healthcare, animal husbandry and agriculture along with evolutionary responses of organisms have resulted in the creation of multi-drug-resistant organisms (MDRO) around the world, in which they can be resistant to most, if not all, currently available antibiotic classes [1]. Drug resistant bacterial infections often lead to increase mortality, the likelihood of hospitalization, prolonged hospital stays, and higher cost of treatment and care. In 2017, the World Health Organization (WHO) listed 12 families of the most problematic pathogens including *Pseudomonas aeruginosa* and *Enterobacteriaceae* (e.g. *Escherichia coli*), *Staphylococcus* spp. especially *S. aureus* for which new antibiotics are urgently needed. Methicillin-resistant *Staphylococcus aureus* (MRSA), vancomycin-resistant enterococci, and MDR Gram negative bacilli, notably *Escherichia coli* and *Klebsiella* species, are the most formidable pathogens and common cause of MDR nosocomial infections [2]. Despite of advanced technology, used in pharma and considerable development in bacterial genomic approaches, antibacterial resistance (AMR) is still responsible for thousands of deaths annually [3]. Therefore, the need for new antimicrobials to combat against pathogens become priority of scientists. AMR is a sophisticated issue, required a multifaceted approach for its talking. Isolating novel natural products (NP) generated by microorganisms is a main source to acquire new chemical for developing antibacterial drugs. Many articles highlight that NPs still play a key role in drugs discovery [4–8]. NPs have a long history of safe and efficient use for development of pharmaceutical products. In addition, natural products provide key scaffolds for development of semi and synthetic drugs. The majority of antibiotics currently used in clinic are derived from microbes [9, 10] which are either naturally occurring products or directly derived from such [7]. Among microbes, actinobacteria in particular *Streptomyces*, are responsible for suppling two-thirds of all known antibiotics utilized in modern medicine [11, 12]. Filamentous *actinomycetes* produce over 64% of the known NP antibiotic classes [13]. Actinobacteria constitute one of the most dominant phyla among bacteria. They are generally free-living, Gram-positive filamentous bacteria with a high GC DNA content ranging from 50% to over 70%, found in aquatic and terrestrial habitats [14]. Many actinobacteria produce mycelia and undergo sophisticated morphological differentiations [15]. Microbial competition, nutritional status, and physical pressures from the natural habitat are all important factors in actinomycetes' development and antibiotic production [16, 17]. Scientific literature evidenced that due to generating an unprecedented potential of bioactive compounds with diverse activities [18], *actinobacteria* have many pharmaceutical and medicinal functions [6, 7, 19]. This encourages researchers to search and screen under-explored or untapped habitats to isolate potential EA strains with antibacterial activities [20, 21]. Exploring new habitats having microbial diversity provide a great potential for discovery of novel bioactive molecules [19]. Numerous studies have linked the antibacterial effect of the herbal plants to the beneficial EA [22, 23]. EA belong to heterogenous group of actinobacteria. Endophytes are microorganisms that live inside plant tissues and have no harmful effects on the host plants. The search for bioactive compounds from underexploited habitats increases the chance for the isolation of desirable species. Therefore, we shifted our research to an untapped habitat; *Citrullus colocynthis* (L.) Schrad*,* for the discovery of novel endophytic isolates and their subsequent bioactive molecules. *C. colocynthis*, a well-known medicinal plant belongs to the *Cucurbitaceae* family that is commonly referred to as bitter apple or bitter cucumber in English and known Hendevaneh Abujahl (Abujahl watermelon) or Kadu Hanzal (bitter ground) in Persian [24]. *Citrullus colocynthis* is distributed in some parts of Asia and Africa. *Citrullus colocynthis* (L.) is also an annual plant that grows in the south, center, west and east areas of Iran [25]. The plant *C. colocynthis* has proved to be a potent source for the creation of new drugs to treat severe human diseases include microbial infections [25–27]. *C. colocynthis* has been consumed as traditional remedy for many years. It is believed that different sections of this herbal plant possess antidiabetic, antihyperlipidemic, hypoglycemic, hypolipidemic, laxative, anticancer, anti-inflammatory, analgesic, vermifuge, hair-growth-promoting, antibacterial, insecticide, antimicrobial, purgative, abortifacient, antineoplastic, profibrinolytic, anti-allergic, antiepileptic, fungicidal, and antioxidant activities. The ethanolic extract of dried fruit pulp, seed, and root of *C. colocynthis* exhibited antagonist activity against Gram positive and Gram-negative bacteria, as well as fungal species in a dose-dependent manner [28]. They suggested the extracts could be effective for treating gastrointestinal, skin, and lungs infections in future. The methanol extracts of the plant showed strong antibacterial activity against *Bacillus subtillis*, *Streptococcus pyogenes*, and *Salmonella typhi* [29]. Besides, the aqueous and acetone extracts of *C. colocynthis* Schrad (roots, stems, leaves, and three stages of its fruit and seeds) showed antibacterial activities against all tested bacteria [30, 31]. The high quantities of phenolic compounds were reported in the aqueous and ethanolic extracts of the plant *C. colocynthis*. The plant hold promises for creation of innovative drugs with a wide range of pharmacological activity that might be used to treat a variety of human diseases due to its efficacy and safety [32].

To the best of our knowledge, no study has been published on the population of EA from *C. colocynthis* (L.) Schrad, and this project is the first study, conducted so far. There is no information available about microbial diversity of *C. colocynthis*. Therefore, the aims of this study were to isolate and identify the EA from *Citrullus colocynthis,* and to assess the antibacterial effectiveness of extracts from the isolates against the hospitalised infection bacteria namely *Staphylococcus* aureus, *Pseudomonas aeruginosa*, *Escherichia coli*.

## Material and methods

### Samples collection and authentication

Healthy plant samples of *Citrullus colocynthis* (L.) Schrad were collected from Qasr-e Shirin (34° 31 10 N and 45° 35 15 E), in the western part of Iran, and transported to the laboratory in sterile bags for surface sterilization. The samples were collected in October 2020 and May 2021 to cover seasonal variation. They were identified and authenticated by an expert, Dr. Nastaran Jalilian, at the Kermanshah Agricultural and Natural Resources Research and Education Centre, Kermanshah, Iran and the voucher specimen (10080) was deposited in the Herbarium of RANK. The plants were dissected to stems, leaves, roots, and fruits, surface-sterilized and cultured within 48 h of sampling.

### Surface sterilization

The surface sterilization of plant samples was performed according to Kaewkla and Franco [33]. Briefly, the plant tissues were rinsed with running tap water to remove all contaminations and any physical debris. After natural air drying and rinsing with distilled water three times, the samples were then dissected to roots, stems, leaves, and fruits using a sterile saw/knife, and tweezers. The segments were then chopped with scissors into small pieces of two-five cm lengths and undergone the surface sterilization with various surface sterilizing agents to prevent non-endophytic bacteria and fungi from growing on the surfaces of the samples.

Each sample was first immersed in 0.1% sterile Tween 20 for 5 min. After being treated with 70% ethanol for 5 min, the samples were rinsed with a freshly made 6% sodium hypochlorite (NaOCl) solution. The samples were rinsed ten times with distilled water (D.W) to remove the chemical residues. The samples were then immersed in sterile 10% (w/v) sodium bicarbonate (NaHCO_3_) for 10 min to retard the growth of endophytic fungi [34], and washed three times with sterile double distilled water. Upon sterilization, the samples were air-dried in a sterile laminar air flow.

To ensure the efficacy of the surface sterilization, 100 μL of the last double distilled washed water was cultivated onto ISP-2 media and incubated at 28 ± 2 °C for 1 week. The absence of microbial growth on the culture media after plating the last washing water demonstrated the effectiveness of surface sterilization [35].

### Isolation of actinobacteria

The selection of an isolation medium is critical because it can directly impact on the diversity of endophytic bacteria that may be isolated from plant tissues and, consequently, on the outcome of the experiment [36]. Four different isolation media (Table 1), namely Yeast extract-casein hydrolysate agar (YECD) [37], potato dextrose agar (PDA) [38], Tap water yeast extract (TWYE) [39], and humic acid-vitamin (HV) agar [40] were selected and supplemented with nystatin at final concentration (20 µg/mL) and nalidixic acid (10 µg/mL) to prevent and repress the growth of fungi and Gram-negative bacteria [41, 42]. The surface-sterilized samples were then aseptically cut into 1–2 cm and placed directly on the culture media in triplicate and the plates were incubated at 28 ± 2 °C, and the growth of EA from tissues was daily monitored for 16 weeks. After emerging, different colonies with putative actinobacteria features were introduced and sub-cultured onto international *Streptomyces* Project-2 (ISP2) [43]. Pure cultures were obtained after two to three successive sub-culturing rounds and transferred to fresh isolation media. Given the possibility that each colony resembles a distinct strain [44]. The pure isolated strains were then maintained on ISP2 cultures and used as master plates to establish stock cultures and the isolates stored in Tryptic Soybean Broth medium (TSB) containing 30% glycerol at − 20 °C for further analysis.

**Table 1:** Composition of culture media used to isolate endophytic actinobacteria

Name of culture medium	Components of the culture medium (1 Liter)	References
PDA (Potato Dextrose Agar)	39 gm	[45]
TWYE (Tap Water Yeast Extract Agar)	Yeast extract 0.25 g, K_2_HPO_4_ 0.50 g, Agar 18.00 g	[39]
YECD (Yeast extract-casein hydrolysate agar)	0.3 g of yeast extract. 0.3 g of D. glucose. 2 g of K_2_HPO_4_, and 18 g of Agar	[37]
Humic acid-vitamin B (HV) agar	Humic acid 1 g, Na_2_HPO_4_ 0.25 g, KCl 0.85 g, MgSO_4_.7H_2_O 0.025 g, FeSO_4_.7H_2_O 0.05 g, CaCO_3_ 0.01 g, Agar18 g, Vitamin B 100×, (added after media autoclaved) 1 mL	[40]

### Identification of actinomycetes

Actinobacteria-like colonies were identified after 8–16 weeks of incubation at 28 °C from the original isolation plates. Well-separated actinobacteria-like colonies were picked and sub-cultured on ISP2 and purified using ISP2 medium. They were cultivated at 28 °C for 10 days on ISP2 agar medium. The isolates were identified based on microscopic characteristic and morphological criteria, such as colony features, colour of aerial and substrate mycelia, the colour of culture, pigmentation, and sporulation, following the general guidelines of the International Streptomyces Project.

Typically, light microscopy was used to examine the basic morphology of hyphae and spores, while scanning electron microscope (SEM) (FEI, Quanta 450, America) was used to examine the microscopic structures of hyphae and spores on the surface [46]. Briefly, the isolates were cultured onto ISP2, the aerial mycelium and spores were obtained on a cover slip. Cover slides were cut into one cm pieces and sterilized. The sterile coverslips were inserted into solidified ISP2 at an angle of 45°. The inoculum was spread along with the glass-agar medium interface and incubated at 28 °C for 10 days. During incubation, the organisms grew over the surface of the glass pieces. The cover slip containing organisms was then coated with a film of gold about 150–200 A° thickness and observed under SEM at an accelerating voltage of 25.000–30.000 V for spore surface ornamentation [47]. The spore chain morphology and ornamentation of the potential strains were analysed.

### Culture and preparation of living cell mass (BIOMASS)

Isolated strains were first cultured on ISP2 and then incubated at 28 °C for five to seven days. Microscopic monitoring of stained and unstained (wet mount) bacteria was further applied for identification. The purity of actinomycete colonies, grown on ISP2 plates were inspected directly, using wet mount or Gram staining method under a light microscope (Nikon Eclipse E100). After ensuring their purity and the absence of contamination, loopful of colonies were cultured on Tryptic Soy Agar (TSA) and/or into Tryptic Soy Broth (TSB), at 28 °C for 7–10 days. The broth culture was incubated in a shaker incubator, (HYSC, Korea) at 160 rpm. Then, it was centrifugated with a Hettich 320R, Germany (4000 rpm, 10 min, 4 °C) to separate the mycelia. The bacteria were then used to extract DNA or study their antimicrobial activities [48].

### DNA extraction

With some modifications, genomic DNA was extracted according to Coombs et al. [37]. Initially, 70–100 mg of the colony were placed in a two mL sterile microtube and washed twice with 500 µL of Tris–EDTA (10 mM Tris; 1 mM EDTA, pH 8.0) by vortex and centrifuging (5 min, 5000 RCF). The sample was then suspended in 500 µL TE buffer containing 0.2 mg mL^−1^ of lysozyme, then vortexed/ spin down (3–5 s.). Following a 60-min incubation at 37 °C, 10µL of 1% (w/v) proteinase K and 10 µL SDS 10% were added, and the mixture was incubated in a water bath at 55 °C for 60 min. The tubes were re-incubated at 55 °C for 10 min after adding 100 µL of 5 M NaCl and 65 µL of CTAB/NaCl (700 mM NaCl, 275 mM CTAB). The equal volume of phehol:chlorofornm:isoamyl alcohol (25:24:1) 600 µL was added to the suspension in a fume hood before being left at room temperature for 30 min with intermittent shaking. To pellet the cell debris, the tubes were centrifuged at 12,000 rpm for 15 min using a microcentrifuge Hermle Z216M, Germany, and the supernatant was carefully transferred to a new 1.5 mL tube. The sample was washed with 500 µL chloroform after incubation for 15 min at room temperature (inverse mix every 7–8 min). Following centrifugation at 12,000 RCF for 15 min, the aqueous phase was transferred to a new sterile 1.5 mL Eppendorf tube. 20 µL of RNase (10 mg/mL) was added and incubated at 37 °C for 60 min. Washing with chloroform was repeated. The aqueous phase was then conveyed to a new sterile 1.5 mL Eppendorf tube after centrifugation at 12,000 RCF for 15 min. The supernatant was treated with 1× volume of 3 M sodium acetate and 3× volume of 100 percent cold ethanol and kept in a freezer at − 20℃ overnight. The DNA was then pelleted by centrifugation at 16,000 RCF for 15 min and supernatant was carefully discarded. The pellet was washed twice with 70% ethanol. The pellet was dried by placing the tubes in the heating block at 55 °C for about 10 min with the lids open, or until the pellet was dry. Finally, the pellets were resuspended in 50 µL of injection water and the quality and quantity of genomic DNA extracts were checked at A260nm/280 nm and A260nm/230 nm ratios by Nanodrop 2000 spectrophotometer (Thermo Scientific, USA), and on 1% agarose gel and stored at − 20 °C. Using agarose gel electrophoresis, the quality of the DNA samples was determined.

### Polymerase chain reaction (PCR)

To demonstrate the microbial diversity of samples, the polymerase chain reaction (PCR) is most commonly employed technique in conjunction with universal primers [49]. PCR amplification of the bacterial 16S rRNA gene was performed as described by Coombs and Franco [37] using Mastercycler® nexus, Eppendorf and a set of universal primers 27F 5′-AGAGTTTGATCMTGGCTCAG-3′, 1492R 5′-TACGGGTACCTTGTTACGACTT-3′ synthesized by Metabion, Germany. The PCR products were about 1500 bp.

Briefly, the amplifications were carried out in a total volume of 25 µL reaction mixture, including 12.5 µL of master mix (Sinaclon Cat.No. MM2062), 0.3 μL of each primer (10 pmol/μL), 1 µL of DNA template (50–100 ng), and 10.9 µL of D.W. Then the microtubes were inserted to the PCR device. PCR conditions included an initial denaturation at 94 °C for two min, followed by 30 cycles of denaturation at 94 °C for one min, annealing at 55 °C for one min, and extension at 72 °C for two min. The final cycle was ended by extension at 72 °C for 10 min and then cooled to 4 °C. The device was given a temperature of 4 °C at the end of the cycles to prevent the PCR product from reacting and degrading.

Electrophoresis was performed for 45 min at 70 V, and the gel was then imaged under Gel Doc device (Vilber, France) to evaluate the PCR products. The PCR products were then sent to Macrogen Inc. (Seoul, Korea) for Sanger sequencing.

### Sequencing and phylogenetic analysis

Bidirectional Sanger sequencing was performed by Macrogen (South Korea) using the same sets of primers used for PCR amplification. MEGA X (Molecular Evolutionary Genetics Analysis) software was used to process the nucleotide sequence of the 16S rRNA gene. The 16S rDNA gene sequences were obtained using the BLASTn tool (Basic Local Alignment Search Tool) (http://www.ncbi.nlm.nih.gov/BLAST/Blast.cgi), and then compared to existing sequences of bacteria in the NCBI (National Center for Biotechnology Information) and EzBioCloud databases to estimate similarity percentages [50]. The hits of subjects’ sequences deposited in international nucleotide databases (e.g., GenBank, EMBL, DDJD, etc.) that provide the best match with the query. Multiple DNA sequence alignment of selected 16S rDNA was performed using the ClustalW algorithm in the MEGA X software [51]. The neighbour-joining method was used to create phylogenetic trees.

Isolates were classified according to their taxonomy using 16S rDNA analysis. The phylogenetic tree was created by MEGA X using the maximum-likelihood method, which was based on the Kimura 2-parameter model [52]. The Kimura two-parameter model was used to calculate pairwise distances for the neighbour-joining procedure (Kimura 1980). Strength and reliability (topologies of the neighbor- joining) of the resultant tree was evaluated after a 1000 bootstrap -replicate analysis [53]. *Brevibacterium linens strain DSM 20425* was used as an outgroup strain for tree constructions [54]. The 16S rDNA gene sequences of the isolates were then deposited in the GenBank and their accession numbers acquired. GenBank accession numbers for the resultant sequences are listed in Table 3. The phylogenetic tree was constructed using the neighbor-joining algorithm/method (based on 1000 bootstrap iterations) of nucleotides sequence of 16S rDNA gene.

### Preparation and screening of isolates for antibacterial activity

To test the antibacterial activity of endophytic isolated strains, the isolates were extracted using ethyl acetate and methanol, respectively. Briefly, the pure isolates were grown on TSA medium and liquid TSB (7 days, 28 ± 2 °C). The solid culture (full TSA plate) containing the culture and the agar medium was then cut into small pieces with a sterile scalpel, and placed into 250 mL Erlenmeyer flasks containing 50 mL of ethyl acetate, and shaken for 24 h (200 RPM) at room temperature. The ethyl acetate solution was filtered and the supernatant collected. Then, 50 mL of methanol was added into the flasks, allowing the device to operate for another 24 h under the same conditions, the extract was separated. Both extracts were concentrated under reduced pressure using rotary evaporator (70 RPM, 38 °C) to yield a dry extract. Then, the extracts were stored at − 20 °C until used for evaluation of antimicrobial activity. The TSB cultures were incubated onto a rotary shaker (HYSC, Korea) at 28 °C with 180 rpm for 72 h [55]. The colonies were then harvested by centrifugation at 4,000 G for 10 min to pellet bacterial cells, and extracted by ethyl acetate and methanol, respectively using the same procedure as used for TSA.

### Antimicrobial activity of fractions using diffusion method

The endophytic isolates were primarily examined for their antimicrobial activity against the test bacterial strains including *Staphylococcus aureus*, *Escherichia coli* and *Pseudomonas aeruginosa* using agar well diffusion methods. Initially, 1–2 loops of test bacteria (*E. coli* ATCC 25922, *Staphylococcus aureus* ATCC 25923 and *Pseudomonas aeruginosa* ATCC 27853) were inoculated into TSB media at 37 °C for 24 h. The growth of the cultures was then adjusted to half the McFarland (OD = 0.2) by measuring the optical density (OD) using a Spekol 1500, spectrophotometer at 600 nm (OD600 nm). The antibiotic agar medium No. 1 (AAM-MERCK) was seeded with the test culture (1% V/V) and dispensed into petri dish plates, and 6 mm diameter wells were prepared using a sterile cork borer at regular intervals to make 10 wells/ per plate.

The antibacterial activity of the crude extract was determined using the agar well diffusion method. Each well was loaded with 50 μL of extracts, allowing the wells to dry completely. The plates were then incubated at 37 °C for 24 h. Vancomycin (20 µg/mL) and Imipenem (15 µg/mL *P. aeruginosa* ATCC 27,853 and 1.5 µg/mL *E. coli* ATCC 25,922) were used as positive controls for Gram-positive and Gram-negative bacteria, respectively. Negative controls were wells containing the same volume of methanol or ethyl acetate (50 mL). Each test was done in duplicates and repeated trice. The width of the zone of inhibition around each well was measured and the average recorded in millimetres (mm).

## Results

### Isolation of endophytic actinomycetes

**Fig. 1:**
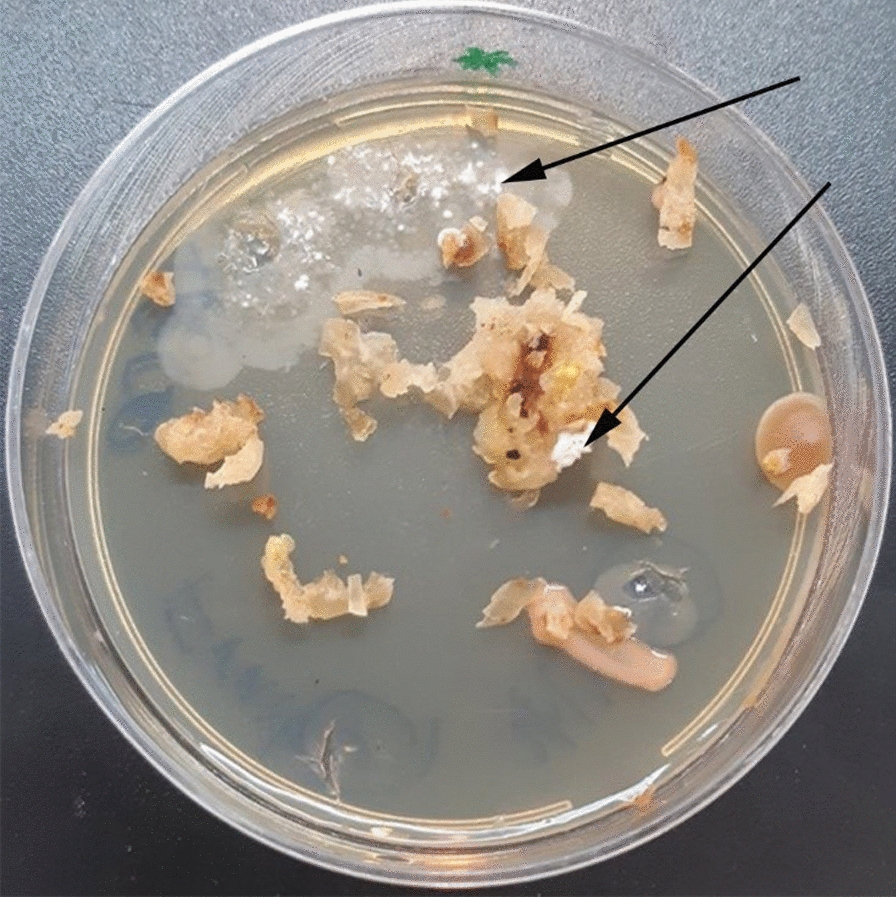
The emerging of actinobacteria after incubation of *Citrullus colocynthis* fruit samples on an YECD medium. The cultures were monitored for 16 weeks. The arrows show that aerial hyphae of *Streptomyces* emerge from surface-sterilized fruit tissues

The surfaced-sterilized samples cultured on four different media (Table 1) and six different actinomycetes strains with various morphological characteristics were isolated and identified based on their morphological characteristics from different part of the plant after 6 weeks of incubation (Fig. 1). This is the first time to record actinobacteria from surface-sterilized *Citrullus colocynthis* plant.

Figure 1 indicates actinobacterial hyphae arising from fruit fragments after 6 weeks of incubation onto YECD medium. The black arrows show the actinobacteria colonies (white mouldy shape) were emerged on YECD medium from the fruit of the plant Abujahl watermelon (*Citrullus colocynthis*). Based on the frequency of the isolation of endophytes, nutrient-poor medium like YECD was the most successful medium for the isolation of EA [56] from *Citrullus colocynthis* leaf tissue.

Six endophytic actinomycetes were isolated from (root, leaf, and fruit) tissues and described based on colonial morphology onto ISP2, ability to generate aerial hyphae and substrate mycelia, and pigmentation as summarised in Table 2. The majority of the 6 isolates (n = 3; 50%) obtained from leaves, followed by fruit (n = 2; 33.3%), and root (n = 1; 16.7%), respectively. Five strains were isolated from the YECD media, one from the TWYE medium, and nil from the PDA and HV media. On the media, the majority of the isolates grew at a moderate to slow rate. The actinobacteria colonies were white, pale yellow in colour after 6 weeks of incubation. All isolates are reported as endophytes for the first time based on morphological, microscopical features and 16S rDNA gene sequencing (Table 2).

**Table 2:** Microscopic and macroscopic characterizations of isolated strains

Identified genus	Color characterization		Microscopic characterization	Identified genus
	Aerial mycelium	Substrate mycelium		
KUMS-C1	Bright white	Yellowish-white	Extensively branched and rod-shaped fragments	*Nocardiopsis*
KUMS-C2	White	white	Extensively branched and rod-shaped fragments	*Nocardiopsis*
KUMS-C3	Creamy	Light yellow	Extensively branched and rod-shaped fragments	*Streptomyces*
KUMS-C4	Dark pink	Pink	Extensively branched and rod-shaped fragments	*Streptomyces*
KUMS-C5	White	Yellow	Extensively branched and rod-shaped fragments	*Nocardiopsis*
KUMS-C6	White-Creamy	Dark brown	Extensively branched and rod-shaped fragments Long chains of spores	*Streptomyces*

### Morphological observations and characteristics

**Fig. 2:**
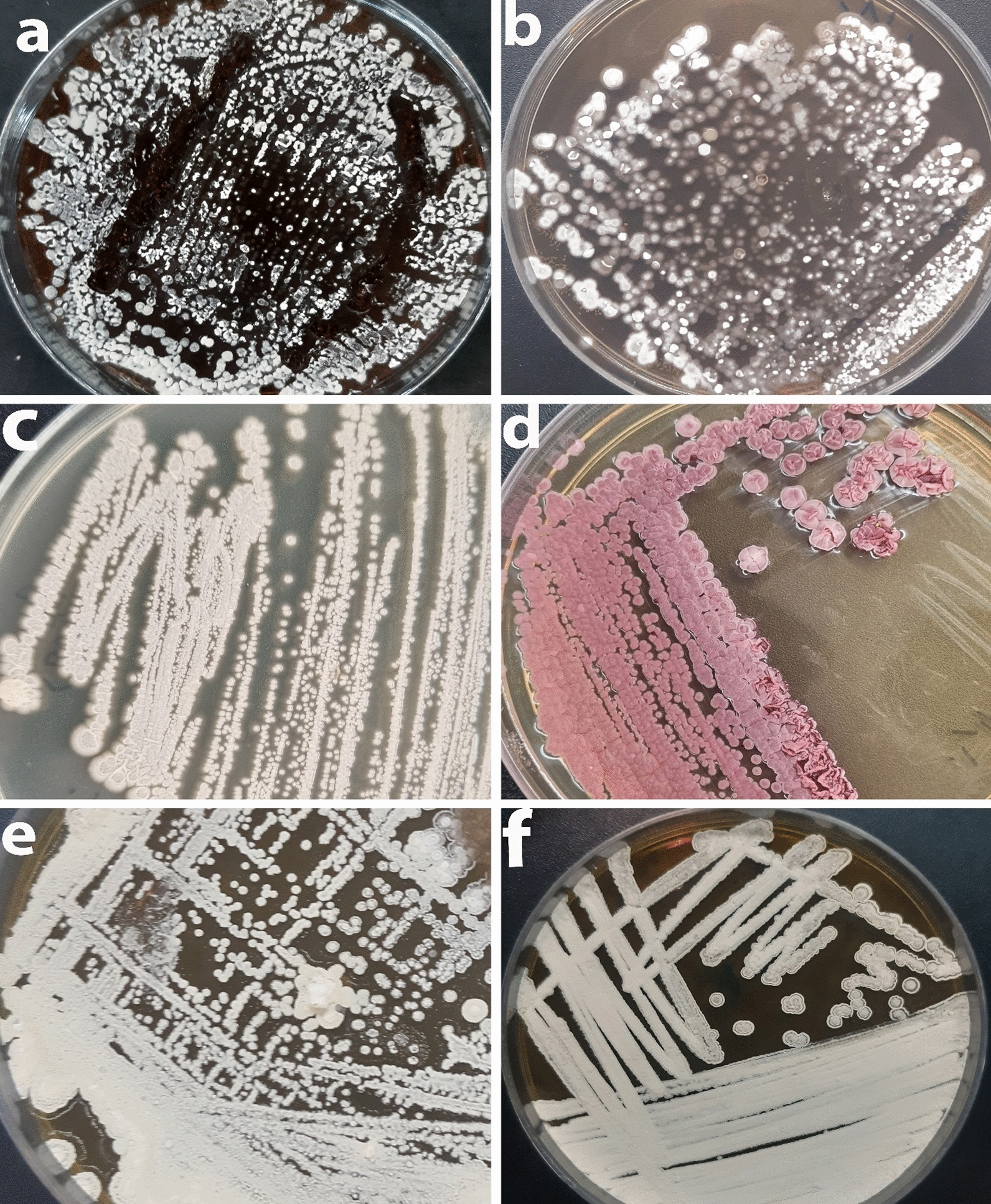
Morphological appearance and feature of six endophytic actinobacteria isolated from *Citrullus colocynthis*, after 1–2 weeks of incubation onto ISP2 at 28 °C. KUMS-C1 isolate; bright white colonies (**a**): KUMS-C2 isolate; spherical white colonies (**b**): KUMS-C3 isolate; creamy-light brownish colonies (**c**). KUMS-C4 isolate; domed dark pink colonies (**d**). KUMS-C5 isolate; creamy colonies (**e**). KUMS-C6 isolate; creamy colonies (**f**)

For isolation, purification and identification of colonies, the isolates were cultivated onto ISP2 and they were categorized based on their morphology. The purified culture was also used as a master plate for storing colonies for further analyses. The isolates were characterized based on their morphological and molecular properties. The cultural and physiological features of isolates were determined using International Streptomyces Project (ISP2) [57, 58]. ISP2-specific media was used to cultivate them for identification. After 7–14 days of incubation onto ISP-2 media, the presence of aerial mycelium, spore mass colour, distinctive reverse colony colour, diffusible pigment, and sporophore and spore chain shape were observed based on the ISCC-NBS centroid colour system [59] as demonstrated in Fig. 2. The rough and leathery texture of typical actinobacteria colonies distinguish them from other bacterial or fungus colonies. The strains KUMS-C1, KUMS-C3 were identified from the fruit tissues, while the strains KUMS-C2, KUMS-C4, KUMS-C5 were isolated from leaf tissue using YECD medium (Fig. 2). The KUMS-C1 (Fig. 2a), strain had dazzling white colonies, but the KUMS-C3 strain had creamy-light brownish colonies (c). KUMS-C2 strain showed spherical white-snowy colonies on ISP2 (b), KUMS-C4 displayed domed dark pink colonies (d), but KUMS-C5 had globular white colonies (e), and KUMS-C6 had the creamy colonies (f) on ISP2 after being isolated from root tissues using YECD (Fig. 2).

**Fig. 3:**
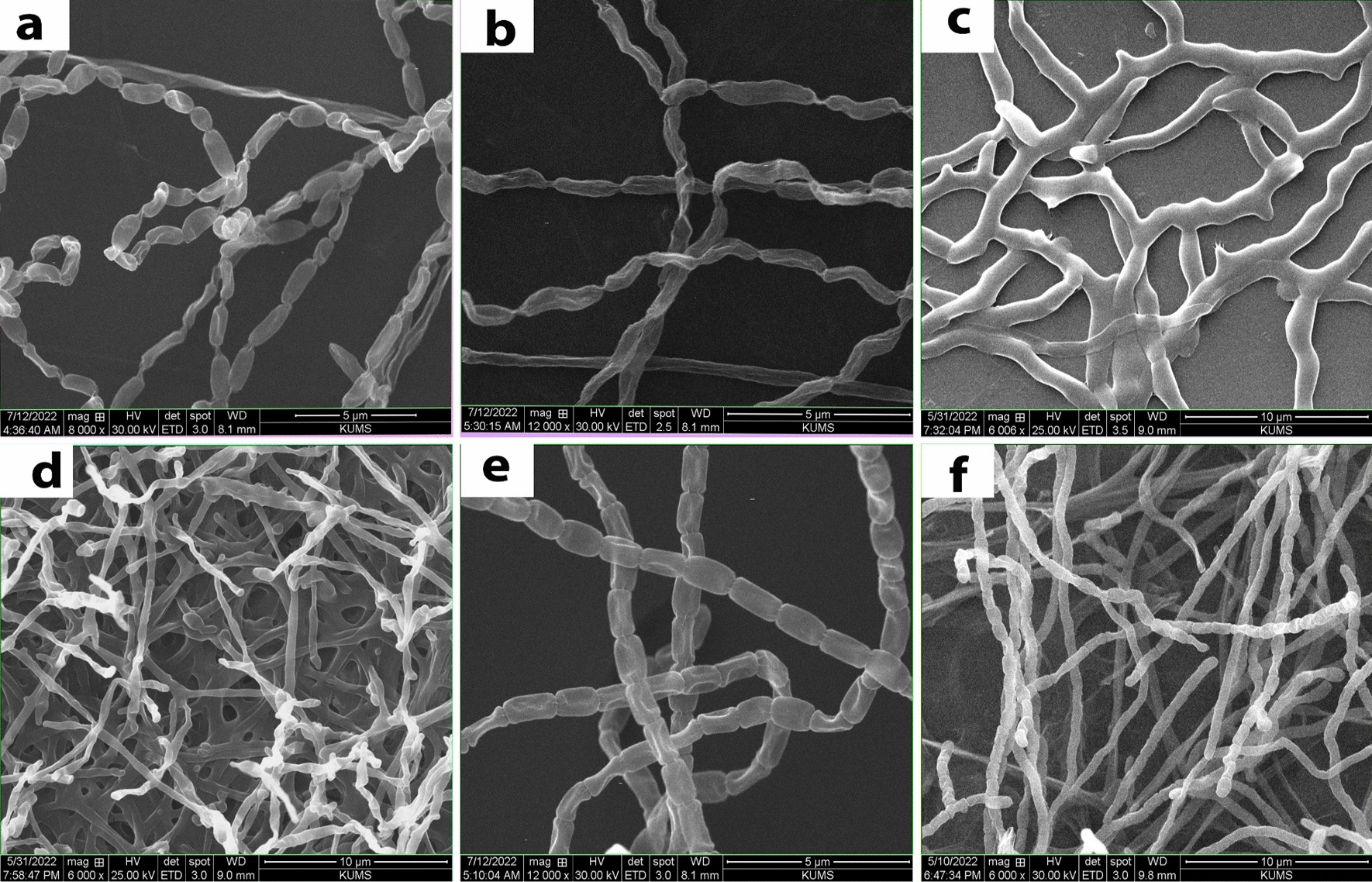
Scanning electron micrograph of mycelia and spores of the isolated strains. The figure shows the spore-chain ornamentation and spore-surface morphology of strains and mycelia after 14 days of incubation on ISP2 medium at 28 °C at different magnifications; KUMS-C1, biconvex cylindrical -shaped spores with smooth surfaces and chain of the spores (**a**); KUMS-C2, long vibrio comma-shaped spores ornamented in long chain of spores (**b**); KUMS-C3, a mesh of branched mycelia structure, not clear septa between the spores (**c**); KUMS-C4, branched mycelia structure with semi spherical spores at the end of the mycelia, oval and ovoid shaped spores (**d**); KUMS-C5, biconvex cylindrical -shaped spores with smooth surfaces, not hairy, long chain of spores (**e**); KUMS-C6, fragmentation of the mycelia creating the spores, cylindrical cube-shaped spores with smooth surfaces, longitudinal chain of spores (**f**)

The morphology of spore chains and mycelia were also studied using scanning electron microscope after 2 weeks growth onto ISP2 medium. Scanning electron micrograph of mycelia and spores of the isolated strains at different magnification after 14 days of incubation on ISP2 medium at 28 °C are shown in Fig. 3. The figure shows the shape and surface of spores, spore-chain ornamentation and mycelia morphologies of strains. The SEM results revealed that most strains produced spores having smooth surfaces. The aerial mycelia generated spores that were cylindrical or cuboid–spiral shapes (Fig. 3); however, the branched and/or comma or rod-shaped and/or long aerial mycelia and spore chains were also observed. Most of the isolates generated biconvex cylindrical shaped spores with smooth surfaces (Fig. 3a, e); however, semi spherical and cube shaped spores (Fig. 3d, f), and a mesh of the branched mycelia were also observed (Fig. 3c).

All isolates were also examined on the basis of 16S rDNA gene and categorised into two families S*treptomycetaceae*, and *Nocardiaceae* with the same ratio.

### 16S rDNA PCR results

**Fig. 4:**
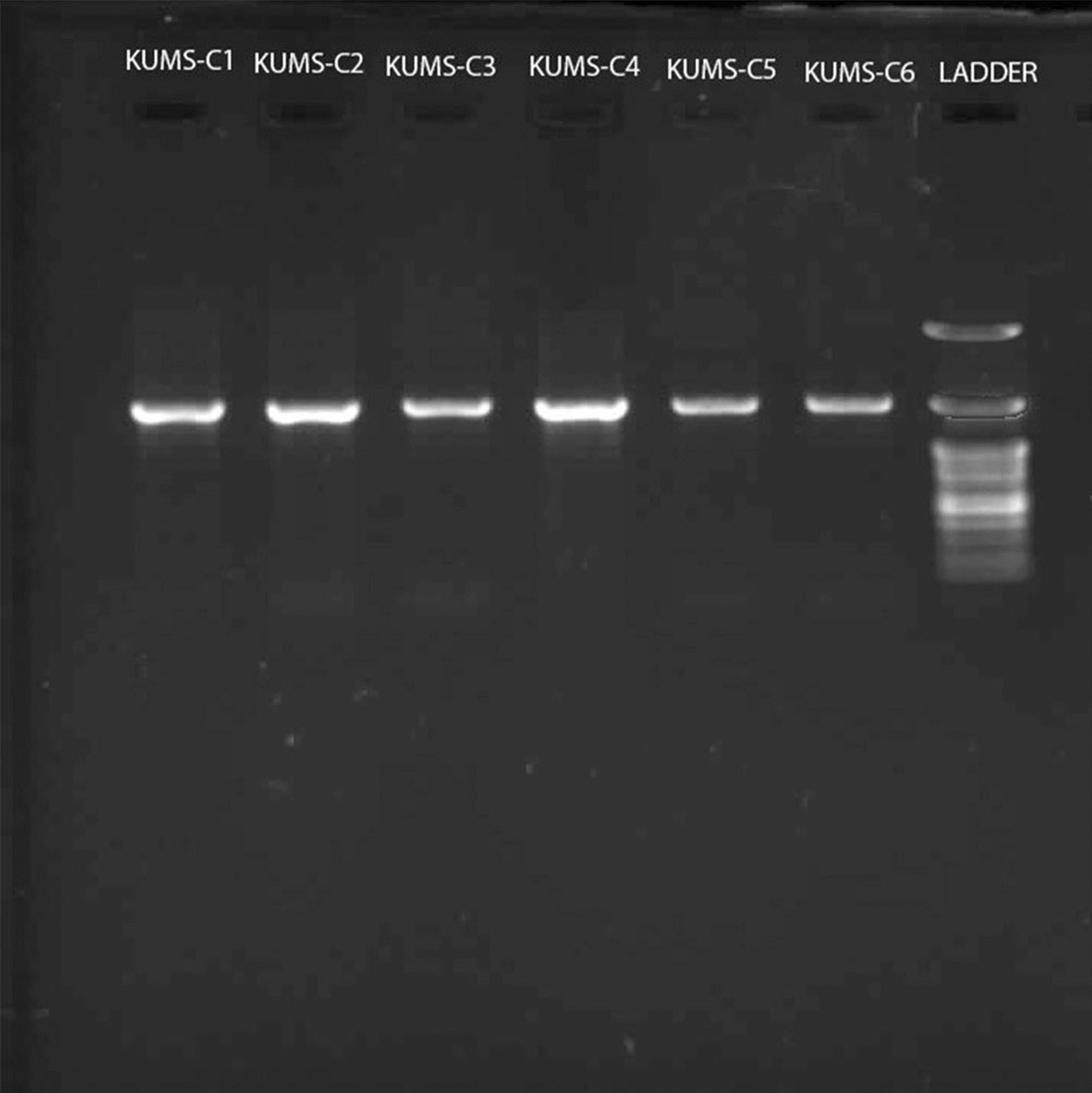
Gel electrophoresis of PCR products using 27F and 1492R primers. The 1.5 Kb amplified 16S rDNA fragments of isolated actinobacteria monitored on agarose gel 1%; from left to right; KUMS-C1 (lane 1), KUMS-C2 (lane 2), KUMS-C3 (lane 3), KUMS-C4 (lane 4), KUMS-C5 (lane 5), KUMS-C6 (lane 6) and Ladder (lane 7)

The genomic DNA was extracted and 16S rDNA amplification carried out. The 16S rDNA fragments were then monitored on a 1% agarose gel electrophoresis as demonstrated in Fig. 4.

### 16S rDNA gene sequencing of isolated actinobacteria

Endophytic actinobacteria strains were identified by sequencing 16S rRNA gene segments. 16S rDNA sequencing data was used to examine the diversity of EA, which was then aligned using BLASTn and the EzBioCloud database. The result of 16S rDNA sequencing was compared with the sequences in the GenBank, NCBI database, and aligned with sequences retrieved from the NCBI GenBank and EzBioCloud database using the ClustalW method to reveal phylogenetic relationships between these sequences and those sequences of bacteria available in dataset and also to determine their similarity values.

**Fig. 5:**
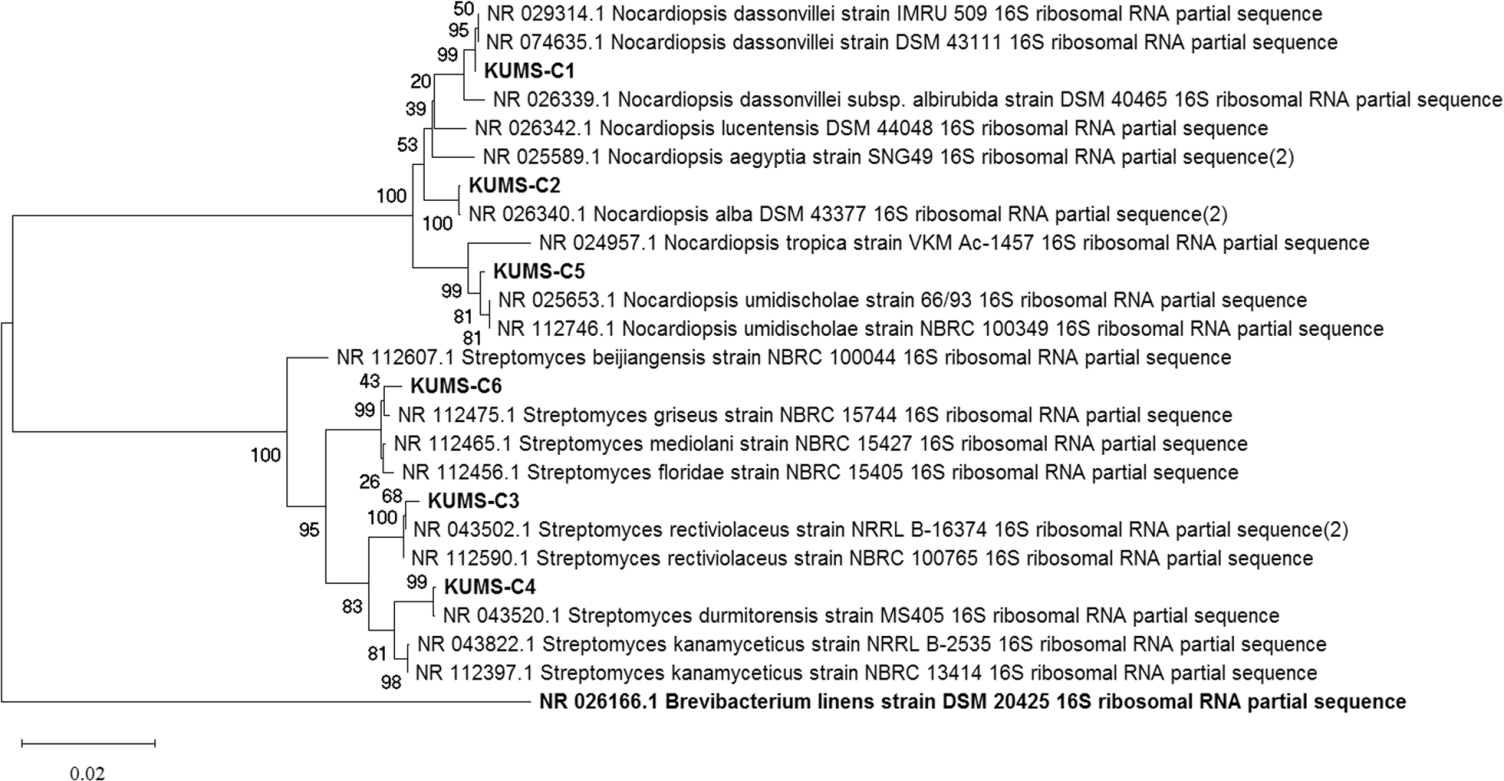
Phylogenetic tree of actinomycetes strains isolated from plant *Citrullus colocynthis*. Phylogenetic tree acquired by Neighbor-joining demonstrating their closely related type strains based on 16S rRNA gene sequencing generated by MEGA X. The numbers at branch nodes show levels of bootstrap support (%) from 1000 replicates and scale bar refers to a phylogenetic distance of 0.02 nucleotide substitutions per site. *Brevibacterium linens *strain DSM 20425 was used as outgroup

Based on 16S rRNA gene alignments and phylogenetic studies of isolates, they were categorized into two main cluster. They all belong to two distinct families within the Phylum *Actinobacteria* (Fig. 5). Strains KUMS-C1, KUMS-C2, KUMS-C5 were located within one clade namely family *Nocardiaceae*, and KUMS-C3, KUMS-C4, KUMS-C6 placed in another cluster, family *Streptomycetaceae*. The phylogenetic tree of the isolates was constructed based on neighbour-joining algorithm. Based on our analyses the similarity of all isolates' 16s rDNA sequences was more than 99.5% when compared to those of data presented in the dataset except for the strain KUMS-C1 with a similarity less than 98.6%. The similarity of the 16S rDNA sequences and their closely related strains in the EzBioCloud database ranged from 98.55% to 99.93%. Among strains, only strain KUMS-C1 had less than 98.65% similarity with their closely related strains, which introduced it as a candidate for being a new strain. All the 16S rDNA gene sequences data have been submitted to the GenBank and acquired corresponding accession numbers are listed in Table 3.

**Table 3:** Identification and similarity of strains isolated from *Citrullus colocynthis*

Isolates	Closest homologs	Family	Pairwise similarity (%)	Tissue of origin	Isolation medium	Accession no
KUMS-C1	*Nocardiopsis dassonvillei* strain IMRU 509	*Nocardiaceae*	98.55	Fruit	YECD	OM980215
KUMS-C2	*Nocardiopsis alba* DSM 43377	*Nocardiaceae*	99.93	Leaf	YECD	OM980216
KUMS-C3	*Streptomyces rectiviolaceus* strain NBRC 100765	*Streptomycetaceae*	99.87	Fruit	TWYE	OM980217
KUMS-C4	*Streptomyces durmitorensis* strain MS405	*Streptomycetaceae*	99.74	Leaf	YECD	OM980218
KUMS-C5	*Nocardiopsis umidischolae* strain 66/93	*Nocardiaceae*	99.72	Leaf	YECD	OM980219
KUMS-C6	*Streptomyces mediolani* strain NBRC 15427	*Streptomycetaceae*	99.73	Root	YECD	OM980220

Identification and similarity of the isolates with their closely related strains according to16s rRNA gene sequence and their identity among selected actinobacteria based on phylogeny trees. The table summarizes the accession no of isolates, origin of tissue, and their families which belong *to Streptomycetaceae* or *Nocardiaceae*

The phylogenetic tree of isolates was built using the neighbour joining tree method, generated by MEGA X, including their closely related strains as shown in Fig. 5. The numbers at branch nodes show levels of bootstrap support (%) from 1000 replicates and scale bar refers to a phylogenetic distance of 0.02 nucleotide substitutions per site. *Brevibacterium linens* strain DSM 20425 was used as outgroup. Besides, their similarities are summarised in Table 3.

The 16S rRNA gene of strain KUMS-C5 was found to have similarity to *Nocardiopsis umidischolae* strain 66/93. The strain KUMS-C**1** was placed with *Nocardiopsis dassonvillei* strain IMRU 509 as the most closely related species with a bootstrap value of 94%**,** while the strain KUMS-C2 was grouped with *Nocardiopsis alba* DSM 43377 as the most closely related species with a high bootstrap value of 100%.

### Antibacterial effect of extracts obtained from mycelium actinobacteria in liquid media by well diffusion method

The set of test bacteria was used to determine the spectrum of antibiotic activity of strains. The antibacterial activity of ethyl acetate and methanolic extracts of six strains obtained from the submerged culture were tested against Gram-positive and gram-negative bacteria including *S aureus* 25923, *P. aeruginosa* 27853, *and E. coli* 25922. Among those, three stains namely (KUMS-C2, KUMS-C3, KUMS-C6) displayed antagonist activity against test bacteria as it is outlined in Table 4. Strain KUMS-C6: closely related to *Streptomyces mediolani* NBRC 15427 showed a wide spectrum of activity. Ethyl acetate extract of strain KUMS-C6 had strong inhibitory effect towards all test bacteria, while its methanolic extract had no inhibitory activity against any of test bacteria. The inhibition zone of this strain against all tested bacteria (*S. aureus*, *P. aeruginosa, E. coli*) ranging from 10.5 to 12.9 mm. Besides, the mycelial Methanolic extract of strains KUMS-C2:closely related to *Nocardiopsis alba* DSM 43377, and KUMS-C3:closely related to *Streptomyces rectiviolaceus* NBRC 100765 were demonstrated antibacterial activity against both *S. aureus*, and, *E. coli*, while their ethyl acetate extracts had no inhibitory activity against test bacteria. While the strains KUMS-C1, KUMS-C4, and KUMS-C5 did not display any inhibition zones against test bacteria, the strains KUMS-C2, KUMS-C3, and KUMS-C6 had inhibitory activity against Gram-positive and Gram-negative bacteria. Noticeably, none of methanolic extracts of strains had inhibitory effect on *P. aeruginosa.*

**Table 4:** The antimicrobial activity of actinobacteria extracts obtained from liquid culture media (TSB) against test bacteria

Ethyl acetate extract				Methanolic extract		
Strain code	*S. aureus* 25923	*P. aeruginosa* 27853	*E. coli* 25922	*S. aureus* 25923	*P. aeruginosa* 27853	*E. coli* 25922
KUMS-C1	0	0	0	0	0	0
KUMS-C2	0	0	0	11.8	0	11.7
KUMS-C3	0	0	0	10.7	0	11.5
KUMS-C4	0	0	0	0	0	0
KUMS-C5	0	0	0	0	0	0
KUMS-C6	11.8	10.5	12.9	0	0	0
Positive control	15.4	10.7	17.7	15.3	10.2	19.3

The diameter of inhibition zones of active extract is recorded in millimeters after incubation at 37 °C for 24 h. Zero means the extract did not have activity towards test bacteria. Imipenem and vancomycin were used as positive controls, while methanol and ethyl acetate were negative controls

### Antibacterial activity of actinobacteria extracts in solid-state culture by well diffusion method

The isolates were cultured on the solid–culture. The antibacterial activity of ethyl acetate and methanolic extracts from solid-state culture of isolated actinobacteria was investigated on test bacteria using well diffusion assay. The results of the antibacterial activity of extracts are summarized in Table 5. The results interestingly indicated that the methanolic extracts from most of strains had inhibitory activity against *P. aeruginosa*, while their ethyl acetate extracts were ineffective towards test bacteria. The methanol extract of KUMS-C1, KUMS-C2, KUMS-C3, and KUMS-C5 were shown antibacterial activity against *P. aeruginosa* by varied potency. Table 5 shows the biological activity of both ethyl acetate and methanolic extracts from endophytic isolates against the test microorganisms.

**Table 5:** Antimicrobial activity of actinobacteria extracts obtained from solid-state culture (TSA) against test organisms

Ethyl acetate extract				Methanolic extract		
Strain code	*S. aureus* 25923	*P*. *aeruginosa* 27853	*E. coli* 25922	*S. aureus* 25923	*P*. *aeruginosa* 27853	*E. coli* 25922
KUMS-C1	0	0	0	0	10	0
KUMS-C2	0	0	0	0	8.4	0
KUMS-C3	0	0	0	0	8.6	0
KUMS-C4	0	0	0	0	0	0
KUMS-C5	0	0	0	0	10	0
KUMS-C6	0	0	0	0	0	0
Positive control	15	8.8	17.9	16	11.6	21.2

All strains showed the zone of inhibition towards *P. aeruginosa* except KUMS-C4, KUMS-C6 strains. None of the strains had antibiotic activity against *S. aureus*, and *E. coli*. Imipenem and vancomycin were used as positive controls, while methanol and ethyl acetate were negative controls

## Discussion

The COVID-19 pandemic has clearly highlighted our world is really brittle to infections that do not respect borders in that has led to the massive use of inappropriate antibiotics, and ultimately will spread the AMR and have negative impacts on human health in the near future. Antimicrobial resistance has currently become one of the pressing major public health problems worldwide. Antibiotic resistance has a substantial global impact, both in terms of economics and clinical morbidity and mortality on human health [8]. The discovery of new antibiotics can reduce the antimicrobial resistance. Although the most effective and cost-efficient technique for reducing AMR is unknown, a diversified strategy is the most likely to succeed this hurdle. Medicinal plant may act as one of the strategies to combat this global issue since they harbour a diverse range of EA. As a result, we chose untapped plant *Citrullus colocynthis* as a source for actinobacteria isolation which are treasure houses for antibiotic productions.

The healthy plant samples were simultaneously inoculated onto four selective media, and six different strains of actinobacteria were isolated and identified from different parts (Root, leaf and fruit) of *Citrullus colocynthis plant*. They were primarily identified using morphological characteristics, as directed by the International Streptomyces Project (ISP2). Endophytic actinomycetes were constantly examined for their appearance and proliferation. They were distinguished by cultural and morphological characteristics, such as the appearance and colour of diffusible pigment and growth (white, white-creamy, bright white, creamy and dark pink). For further identification Gram-straining were also conducted. The isolates were also examined under light and electron microscopes for filamentous nature, spore shapes, spiral sporospores, spores ornamentation, and hyphae [60, 61].

The spore-chain morphology and spore shape and surface ornamentations are vital for identification and classification of actinobacteria [62]. The growth and sporulation rates were varied among the individual isolates. The SEM findings revealed that some isolates had vibrio comma -shaped spores with twisted chain of spores (KUMS-C2), while some strains had a chain of cylindrical or cubed shape spores with smooth (KUMS-C5) and/or indented surfaces (KUMS-C6). To the best our knowledge, this is the first report on the identification of EA form *Citrullus colocynthis* (L.) Schrad and their antibacterial activity.

Then, the morphological and molecular analyses of strains revealed that they belong to two families namely *Streptomycetaceae* and *Nocardiaceae*. They were identified from different sections of plants. KUMS-C1 and KUMS-C3 closely related to *Streptomyces rectiviolaceus* strain NBRC 100765 were isolated from fruit and KUMS-C2 strain closely related to *Nocardiopsis alba* DSM 43377, KUMS-C4 related to *Streptomyces durmitorensis* strain MS 405 and KUMS-C5 related to *Nocardiopsis umidischolae* strain 66/93 were isolated from leaves and KUMS-C6 closely related to the *Streptomyces mediolani* strain NBRC 15427 was identified in the root part, no strain was isolated from the stem. The result revealed that the leaves are a good habitat for EA. Plant species, genotype, tissue, development stage, and environmental circumstances can all influence the total population density of endophytic bacteria found in plants. The bacterial spectrum, on the other hand, appears to be the result of niche specialization, variations in colonization pathways, or mutual exclusion across bacterial populations [63]. Four media including YECD, PDA, HV, and TWYE were used to isolate potential actinobacteria. Five strains were isolated from YECD, one strain from TWYE and none from HV and PDA. This result indicated that YECD was the most suitable and successful medium for isolating endophytic actinomycete followed by TWYE. It was documented that the ingredients of isolation media seems to be more influential than the incubation time in yielding actinobacteria even in the presence of antifungal drugs and surface sanitation [37, 64]. No growth of actinobacteria was detected on PDA and HV media. Before fungi could colonize the plate and hide the existence of actinobacteria, the low nutrient media (YECD and TWEY) stimulated the emergence of actinobacteria from plant pieces which the same result was concluded by Coombs, Franco [37]. Unlike previous study, the high percentage of isolates were from leaves. Fifty percentage of strains were isolated from leaves and the other 50% isolated from fruits and root of the plant. Despite of applying different strategies including using four isolation media and a prolong incubation time of the isolation cultures, the number of isolates was less than whatever we expected. This result might likely be due to the intrinsic feature of plant species which are herbaceous, not woody plants and are more vulnerable to the surface sterilization chemicals and thus suggested that these substances might have penetrated to the samples and perished EA or they might be unculturable on the selected media.

The phylogenetic analysis revealed the relationship between the isolated strains and those bacteria exist in the NCBI datasets. Based on the 16S rRNA sequencing, KUMS-C3, KUMS-C4 and KUMS-C6 strains were clustered together in three genera of *Streptomyces,* whereas KUMS-C1, KUMS-C2 and KUMS-C5 strains were grouped into *Nocardiopsis*. KUMS-C1 has 98.55% similarity with *Nocardiopsis dassonvillei* strain IMRU 509 which constitutes as a new species/ strain as a result the name *Nocardiopsis colocynthis* sp is proposed for the isolated strain. The strains KUMS-C2 to KUMS-C6 have (99.93%, 99.87%, 99.74%, 99.72%, 99.73%) identity with their closely related strains (*Nocardiopsis alba* DSM 43377, *Streptomyces rectiviolaceus* strain NBRC 100765, *Streptomyces durmitorensis* strain MS405, *Nocardiopsis umidischolae* strain 66/93, *Streptomyces mediolani* strain NBRC 15427), respectively.

Our result was consistent with findings reported by Shutsrirung and Chromkaew [65] who found that *Streptomyces* sp. and *Nocardiopsis* sp. were most common endophytic actinomycetes isolated, followed by *Microbispora* sp.. Sequence analysis by Tian, Cao [66] were used to identify 38 representative isolates' 16S rRNA genes (1.25 kb). *Streptomyces* spp. and *Nocardiodies* sp. were isolated from rice stems and roots, with *Streptomyces* being the most often isolated species. Wei and co-workers isolated a number of actinobacteria from the leaf of tea plants, in which the majority were non-*Streptomyces* sp [67], while in our study half of strains were of *Streptomyces* sp.

The antibacterial activity of isolates was assessed against test bacteria namely *S*. *aureus, E. coli* and *P. aeruginosa* using both solid and broth culture states. Some strains showed antagonist activity against both Gram-positive and Gram-negative bacteria in submerged culture; however, in solid culture, the methanolic extracts had only antagonist activity against *P. aeruginosa.* The highest level of antibiotic activity of extracts was observed in the submerged state culture as compared to solid- state. The ethyl acetate extract of isolate KUMS-C6 showed a broad spectrum of antibiotic property against all tested bacteria in different ranges, and demonstrated the largest inhibition zone against *E. coli* 25922 as compared to all isolated strains. Thus, this strain could be a potential candidate for production of new drug compounds in the future. The antibacterial compounds generated by this strain have probably nonpolar structures. Ethyl acetate extract of the broth culture of KUMS-C6 displayed a larger zone of inhibition against *E. coli.* Likewise Conti, Chagas [68] found that EA isolated from medicinal plant *Lychnophora ericoides* had anticancer, and antibacterial properties*.*

The mycelial metabolic extracts of broth culture of KUMS-C2 and KUMS-C3 showed antagonist activity against both Gram-positive and Gram-negative bacteria namely *S.* aureus and *E. coli,* while they had no antibacterial activity against *P. aeruginosa*. This might be resulted from the penetration of polar bioactive compounds to the test organisms.

The ethyl acetate extract of KUMS-C2 and KUMS-C3 had no effects on test bacteria, in which the variations in total phenolic, phenolic acid and total flavonoid content between the methanol and ethyl acetate extracts might be attributed to the discrepancy in activity [69]. This discrepancy in sensitivity might be attributed to morphological differences such as outer membrane of *E coli* having lipopolysaccharide which makes the cell wall impermeable to lipophilic extracts, Lipid Transfer Proteins (LTPs) and the structure of pores and efflux pumps or it might be likely due to functionally difference in the PhoP-PhoQ systems of *E. coli* and *P. aeruginosa*.

In solid state culture, interestingly, the methanolic extracts of all *Nocardiopsis* sp. and *Streptomyces rectiviolaceus* exhibited inhibitory activity towards *P. aeruginosa*, which clearly highlights the ability of those for producing antibiotics. This propriety resulted from the production of polar bioactive compounds. It is noticeable that the methanolic extracts of KUMS-C2 and KUMS-C3 showed antagonist activity against test bacteria in both submerged and solid-state cultures. It seems that the methanolic extracts showed more antimicrobial activity than ethyl acetate extracts.

Rajivgandhi and Ramachandran [70] investigated antimicrobial activity of 20 isolated endophytic strains against uropathogenic multidrug-resistant bacteria. Ameen et al. [71] screened thirty endophytic actinobacterial strains isolated from wild medicinal plants for potential biocontrol agents against antibiotic-resistant mastitis bacteria. Ethyl acetate extracts of actinobacteria were inhibited the growth of *S. aureus*, methicillin-resistant *Staphylococcus aureus* (MRSA), *E. coli*, and *P. aeruginosa*. KUMS-C6 shows 99.73% similarity with *Streptomyces mediolani. Streptomyces mediolani* sp. AC37 was isolated as an endophytic bacteria by Jiménez et al. [72] from the root system of *Taxus baccata* and generated (-)-8-*O*-methyltetrangomycin as a metabolite. Mutation was employed to induce genetic variations in order to select isolates that produced more (-)-8-*O*-methyltetrangomycin which is an angucycline antibiotic. It was reported that *Nocardiopsis dassonvillei*, a closely related strain to KUMS-C1, demonstrated antibacterial activity against a panel of pathogens including *P. aeruginosa* [73] which is in agreement with our findings that KUMS-C1 extracts had activity against *P. aeruginosa*. α-pyrone isolated from *N. dassonvillei* HDN 154151 showed antibacterial activity [74]. Different subspecies of *N. dassonvillei* have been reported from marine sponges, marine sediments and puffer fish, generating several bioactive and antibiotic compounds [75]. The interaction of this endophytic actinomycetes (*N. dassonvillei*) with aerial sections of *Miconia albicans* was originally described by Piza et al. [76]. They reported the isolate has antagonist activity against significant clinical pathogens. Strain HT88 was isolated from *Mallotus nudiflorus* L. fresh stems and identified as *Nocardiopsis* sp. by Xiang et al. [77], which showed 100% identity to *Nocardiopsis alba*. Molecular analysis revealed that KUMS-C2 is closely related to *Nocardiopsis alba* with 99.93% similarity. Different subspecies of *N. alba* were reported from marine sponges, honeybees and mollusk, producing substances which had a broad spectrum of antimicrobial activities [75]. For example, HT88 extracts showed antimicrobial activity against test bacteria which is in accordance with our findings; antimicrobial activity of KUMS-C2. Two antibacterial cyclic dipeptides, cyclo(ΔPhe-ΔLeu) (albonoursin) and cyclo(ΔmTyr-ΔLeu), reported from this strain [78] which is in line with our antibacterial data for the closest isolate. KUMS-C4 was closely related to the *Streptomyces durmitorensis* strain. A novel polyene macrolide family, 32,33-didehydroroflamycoin, reported from *S. durmitorensis* which induces cell death in various cancer cell lines [79]. Previous studies concluded that antimicrobial compounds produced by actinobacteria might aid in developing novel antibiotics and better ways for preventing the spread of antibiotic-resistant infections [80–82]. The medicinal plant is regarded a rich source of actinobacteria with antibacterial activity, as many earlier reports have stated [83–89].

In general antibiotic assay revealed that the metabolic extract of all strains except KUMS-C4 and KUMS-C6 had inhibitory effect on *P. aeruginosa*, while ethyl acetate extract of all strains showed no zone of the inhibition against test organisms. In the solid-state culture, the inhibition zone of extracts and antibiotic activity was slightly lower than submerged state. This discrepancy might be due to the presence of the amount of oxygen dissolved in the culture.

Some species of *Streptomyces rectiviolaceus*, closely related to KUMS-C3, was reported to have inhibitory activity against various plant pathogenic fungi [90]. It was documented that *S. rectiviolaceus* DY46 produces 32,33-didehydroroflamycoin having antifungal activity. *Streptomyces durmitorensis* strain MS405, closely related to KUMS-C4, was originally isolated from soil [91], and this is the first time to be reported as an endophyte. KUMS-C5 is also reported the first time as endophytic actinobacteria*. Nocardiopsis umidischolae* strain was firstly reported in water-damaged indoor environments of a moist school. To our knowledge this is the first time to report the strains KUMS-C3, KUMS-C4, and KUMS-C5 as new endophytic actinobacteria.

## Conclusion and future perspectives and directions

New antibiotics are disparately needed to combat MDR bacteria. Actinobacteria are reported to be primary contributors to the antibiotic discovery in the last century. We have reported six EA belonging to two different families; *Nocardiaceae, Streptomycetaceae* from various tissues of plant *Citrullus colocynthis*, for the first time. These natural metabolites possess a diverse chemical structure with a wide spectrum of biological functions and hold promise for a variety of pharmaceutical and biotechnological applications. Microbial natural products due to having unique physicochemical characteristics are still remained as primary sources for the discovery of new antibiotics as they have unique properties although new approaches should be applied to improve their efficiency. Due to the alarming rates of bacterial resistance to antibiotics, these natural products have become crucial to reverse the game against bacterial resistance.

To our knowledge, this is the first report on the diversity and antimicrobial activity of EA isolated from *C. colocynthis*. Our study has addressed that the medicinal plant *C. colocynthis* harbours a considerable number of EA, producing a wide renege of natural bioactive compounds exhibiting a broad-spectrum of antibacterial activity. Five isolated strains had antagonist activity against *S. aureus*, *P. aeruginosa*, *E. coli.* Systematic research is also needed to determine the chemical diversity of bioactive metabolites produced by these endophytes, which could lead to the discovery of novel natural products for the treatment of bacterial pathogens. In addition, more research is required to determine the best conditions for optimum production of active components.

Based on phylogenetical data presented, the strain KUMS-C1 constitutes a novel species of the genus *Nocardiopsis* which requires further molecular analysis including whole genome analysis. Most of isolated strains are presented adverse activity against test bacteria in which more genomic analyses of their biosynthetic gene clusters and polyphasic study are needed.

Further chemical profiling study is required to purify and elucidate the structure of antibacterial bioactive compounds from the extracts in order to develop putative drugs against resistant bacteria using high-throughput tools such as HPLC, MS and NMR. Many of the biosynthetic gene clusters for antibiotics are poorly expressed under laboratory conditions [92], but they are likely expressed in response to host-specific demands. Although there are many ways to activate the silent BGCs in actinobacteria, applying new tools and techniques such as CRISPR-Cas9 genome editing are probably led to discovery of new antibiotics [13]. We ensure that the future of antibiotic discovery is bright since new technologies such as genome mining and editing are established to discover new bioactive natural products.

It is hoped that by identifying and applying the appropriate combination therapies, a step can be taken to overcome concerns about nosocomial infections and antibiotic resistance resulting health-economic burden. Therefore, future efforts should focus on further purification and examination of the bioactive compounds from crud isolated extracts and their efficacy to produce drug leads and/or biologically active substances, and medicinal products to improve human health.

## Data Availability

The following accession numbers have been assigned to the nucleotide sequences generated during the current study, deposited in the GenBank databases: OM980215, OM980216, OM980217, OM980218, OM980219 and OM980220.
